# A joint individual-based model coupling growth and mortality reveals that tree vigor is a key component of tropical forest dynamics

**DOI:** 10.1002/ece3.1532

**Published:** 2015-05-29

**Authors:** Mélaine Aubry-Kientz, Vivien Rossi, Jean-Jacques Boreux, Bruno Hérault

**Affiliations:** 1 UMR 'Ecologie des Forêts de Guyane', Université des Antilles et de la GuyaneCampus agronomique de Kourou, Kourou, France; 2 UMR 'Ecologie des Forêts de Guyane', CIRADCampus agronomique de Kourou, Kourou, France; 3 UPR Bsef, CIRADMontpellier, France; 4 UMMISCO (UMI 209), Université de Yaoundé IBP337, Yaoundé, Cameroun; 5 Département des Sciences et Gestion de l'environnement, Université de LiègeArlon, Belgium

**Keywords:** Bayesian framework, estimation method, individual-based model, linked models, MCMC, Paracou, tropical forest dynamic

## Abstract

Tree vigor is often used as a covariate when tree mortality is predicted from tree growth in tropical forest dynamic models, but it is rarely explicitly accounted for in a coherent modeling framework. We quantify tree vigor at the individual tree level, based on the difference between expected and observed growth. The available methods to join nonlinear tree growth and mortality processes are not commonly used by forest ecologists so that we develop an inference methodology based on an MCMC approach, allowing us to sample the parameters of the growth and mortality model according to their posterior distribution using the joint model likelihood. We apply our framework to a set of data on the 20-year dynamics of a forest in Paracou, French Guiana, taking advantage of functional trait-based growth and mortality models already developed independently. Our results showed that growth and mortality are intimately linked and that the vigor estimator is an essential predictor of mortality, highlighting that trees growing more than expected have a far lower probability of dying. Our joint model methodology is sufficiently generic to be used to join two longitudinal and punctual linked processes and thus may be applied to a wide range of growth and mortality models. In the context of global changes, such joint models are urgently needed in tropical forests to analyze, and then predict, the effects of the ongoing changes on the tree dynamics in hyperdiverse tropical forests.

## Introduction

The biological processes responsible for tree mortality involve a combination of environmental stresses, but early warning signs can be detected by looking at the behavior of tree growth (Pedersen 1998; Dobbertin 2005). Indeed, trees exhibiting the highest growth rates have a better chance to stay alive, while trees with lower than expected growth rates are more likely to die before their expected size at maturity (Chao et al. 2008). This phenomenon is often called tree vigor, a good starting point from which to build coupled models of growth and mortality that explicitly take into account the biological link between these two processes.

Few studies conducted in tropical forests have proposed a clear quantification of vigor, which is a concept rather than a measurable quantity. Shigo (2004) proposed a definition of tree vigor as “the capacity to resist strain; a genetic factor, a potential force against any threats to survival” and distinguished between vigor and vitality: “the ability to grow under the conditions present.” Most studies have not distinguished between these two views, and tree vigor has been generally related to high survival or high growth rates. Indeed, on the one hand, this concept may be related to tree mortality, as a loss of vigor is expected to imply a higher susceptibility to stresses (Manion 1981). On the other hand, vigor may be linked with growth, and most studies using the term vigor have described this quantity as the diameter growth rate (Buchman et al. 1983; Rosso and Hansen 1998). A more complex estimator of vigor based on growth is the stem growth per unit of leaf area, which was used in Waring et al. (1980) as an estimator of the proportion of carbon allocated to stem wood production. Based on growth rates, this kind of vigor estimator may then be linked with mortality, and past growth may be included as a predictor in mortality models (Bigler et al. 2004; Chao et al. 2008). Vigor may also be related to the pressure undergone in tree dynamics, such as competition and environmental stress. For instance, vigor was sometimes defined as competitive vigor, the quality of how a tree is able to compete for resources, or it may also be used as capability to react to environmental stresses.

One can also consider that tree vigor is an individual property that describes the health of the tree and its capacity to maintain this healthy state, independent of not only the processes observed (growth, mortality, competition, etc.), but also the species strategies and the ontogeny. Indeed in tropical forests where species diversity is high growth strategies highly differ between species (Flores et al. 2014). Pioneer species have high growth rates, while many other species grow slowly. A good vigor estimator has to be independent of the species identity, in the knowledge that an individual having a low growth may effectively have very low vigor if it belongs to a species that normally grows fast, but a normal vigor value if it belongs to a slow-growing species. Moreover, growth is highly dependent on the ontogenetic stage. Most tree species attained the maximum growth rate at intermediate diameters and showed hump-shaped ontogenetic growth trajectories (Hérault et al. 2011). The probability of mortality is also strongly dependent on ontogenetic stages. Young trees and old trees die more frequently, because of the intense competition among the youngest and senescence of the oldest (King et al. 2006; Muller-Landau et al. 2006). Thus, a good vigor estimator also has to be independent not only of the species ecological strategy but also of the ontogenetic stage.

In this study, we (1) propose a quantification of tree individual vigor based on the difference between the computed expected growth and the growth observed in the field; (2) present a way to bind growth and mortality processes in a single modeling framework and a way to select the useful covariates in the joint model and (3) report the gain in predicting mortality rates when accounting for tree vigor. We then discuss the possibilities that such a methodology offers, as well as limitations and possible improvements.

A wide range of linear models is used to describe the tree growth process because linear models are easy to implement and infer using user-friendly available tools. However, as our knowledge on the growth process has improved, diverse nonlinear models are being developed in order to take into account the size-dependent growth trajectory (Paine et al. 2012) as it is generally acknowledged that most tropical tree species attain maximal absolute growth rates at intermediate sizes (Hérault et al. 2011), leading to a typical hump-backed, growth–diameter relationship. During the early stages of tree growth, the growth curve of the tree size accelerates rapidly because taller trees have better access to light and a larger photosynthetic area (Sterck et al. 2003), while the growth rates of mature and old trees often decline because of (1) resource reallocation toward reproduction (Thomas 1996), (2) respiration costs of roots and stems becoming too high (Ryan and Yoder 1997), or (3) trees beginning to senesce. Nonlinear models have to be inferred using likelihood maximization or Bayesian Monte-Carlo Markov chain (MCMC) methods, implying that building a sophisticated nonlinear model requires more computational effort for parametrization [e.g., Rüger et al. (2012)]. Paradoxically, while the biological determinants of mortality seem less well known than those of growth, mortality modeling may be simpler because it is a binary process that is well captured by logistic models (Monserud and Sterba 1999; Brando et al. 2012; Ruiz-Benito et al. 2013). In this study, we used an individual tree growth and mortality model, parameterized at the community level, both of which use functional traits and ontogenetic stage as predictors of tree dynamic. Both models were formerly proved to be efficient predictors of forest dynamics in hyperdiverse tropical forests (Hérault et al. 2011; Aubry-Kientz et al. 2013).

## Materials and Methods

### Data

The study was conducted using data from the Paracou experimental site (5^∘^18'N, 52 ^∘^55'W), a lowland tropical rain forest near Sinnamary, French Guiana. The forest is typical of Guianan rain forests, with dominant tree families including Fabaceae, Chrysobalanaceae, Lecythidaceae, and Sapotaceae and with more than 500 woody species attaining 10 cm diameter at breast height (DBH). Mean annual precipitation averages 2980 mm (30-year period), with a long dry season from mid-August to mid-November and a short dry season in March (Wagner et al. 2011).

Two data sets were used in the study. The first data set is a 20-year inventory of all trees >10 cm DBH in six natural forest plots of 6.25 ha. Censuses of mortality and diameter growth were conducted every 10 years. DBH was calculated from precise measurements of circumferences at 0.5 cm. We excluded individuals with buttresses or other problems that required an increase in measurement because we were unsure about the height of the initial points of measurement on these trees. The data set contained 17,151 trees. For each tree every 10 years, we know the location, DBH, vernacular name, and status (dead or alive). The vernacular name is the name used by local tree spotters. The botanical determination of the trees was completed in 2012, following extensive inventories with voucher collections and determination at regional and international herbaria. Hence, a large number of the trees that died during the study period (1991–2011) have no botanical determination, only a vernacular name (Guitet et al. 2014).

The second data set was a collection of six functional traits of the 335 Guianan tree species that occur at the Paracou site (Table1). Traits are related to leaf economics, stem economics, and life history and are extracted from a large database (Baraloto, et al. 2010a,b). Some of these functional traits are accurate proxies of growth trajectories (Hérault, et al., [Bibr b15],[Bibr b16]) and mortality rates (Aubry-Kientz et al. 2013). The set of data on traits is not complete for all individuals because all trees were not determined at the species level, and some trait values were not available for all species. We used the method of Aubry-Kientz et al. (2013) to compute the posterior distribution of the traits for each individual.

**Table 1:** The six functional traits used in the study

Functional traits	Abbreviation	Range
Maximum diameter (m)	*DBHmax*	[0.13; 1.11]
Maximum height (dm)	*Hmax*	[0.8; 5.6]
Stem and branch orientation (orthotropic (1); plagiotropic (0))	*Ortho*	–
Trunk xylem density (g cm^−3^)	*WD*	[0.28; 0.91]
Laminar toughness (N)	*Tough*	[0.22; 11.4]
Foliar *δ*13*C* composition (^0^/_0_)	*δ*13*C*	[−3.61; −2.62]

For each functional trait, variable name, unit, abbreviation used in the article, and range of values.

### Growth and mortality models

The growth individual-based model is a nonlinear model developed by Hérault et al. (2011) where functional traits and ontogenetic stages of the trees are explicit predictors of the growth trajectory.

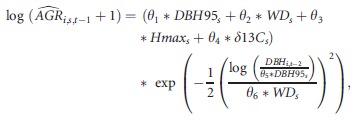
and

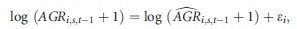
with

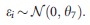

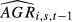
 is the predicted growth between time *t*−2 and time *t*−1; *DBH*95_*s*_, *Hmax*_*s*_, *Tough*_*s*_ and *δ*13*C*_*s*_ are functional traits of species *s* to which tree *i* belongs (Table1); *θ*_1_, *θ*_2_,…*θ*_7_ are the parameters to be estimated; and *ɛ*_*i*_ is an individual error term following a normal distribution.

The mortality individual-based model was developed by Aubry-Kientz et al. (2013) to compute the individual probability of dying at each time step. At each time step, a tree *i* of species *s* may die with probability *p*_*i*,*s*,*t*_.

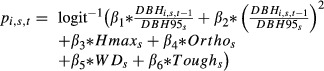
*DBH*95_*s*_, *Hmax*_*s*_, *Ortho*_*s*_, *WD*_*s*_, and *Tough*_*s*_ are functional traits of species *s* to which tree *i* belongs (Table1); *β*_1_, *β*_2_,…*β*_6_ are the parameters to be estimated.

### Vigor quantification

Ideally, a vigor estimator should be independent from the ontogenetic stage 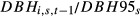 and from the species ecological strategies to be quantified at the individual tree level. These two factors are included in the growth model so that we estimate vigor as the difference between the computed expected growth and the growth observed in the field, 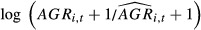, where *AGR*_*i*,*t*_ is the observed growth between time *t*−1 and time *t* for tree *i*, and 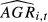 is the predicted growth between time *t*−1 and time *t* for tree *i* using the growth model. *A priori*, we had no reason to work with a metric other than the linear difference (or ratio in logarithm). If the difference is zero, then the vigor is zero that means that the observed growth does not affect the likelihood of dying. If the predicted growth is higher than the observed growth, then the vigor is negative and vice versa.

### Coupling growth and mortality

The growth and mortality processes were linked through tree vigor and are parametrized simultaneously. If tree *i* stays alive, it grows at a growth rate *AGR*_*i*,*s*,*t*_, and its diameter *D*_*i*,*t*−1_ becomes *D*_*i*,*t*_. The joint model likelihood is then

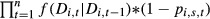
 if tree *i* stays alive during the length of the study period,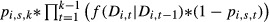 if tree *i* dies between time *k*−1 and time *k*

where

- *f*(*D*_*i*,*t*_|*D*_*i*,*t*−1_) is the probability density for a tree with diameter *D*_*i*,*t*−1_ at time *t*−1 to have a diameter *D*_*i*,*t*_ at time t; this quantity is used to compute the vigor estimator
- *p*_*i*,*s*,*t*_ is the probability of dying between time *t*−1 and time *t*, which depends on the vigor estimator 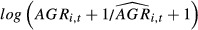, added as a new predictor in the mortality model by multiplying the vigor estimator by *β*_0_ to give

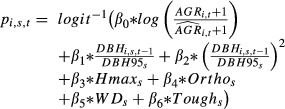

The model computes the mortality probability *p*_*i*,*s*,*t*_ and the predicted growth rate 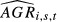. In this study, these two demographical parameters are computed using a joint model inspired by the studies of Hérault et al. (2011) and Aubry-Kientz et al. (2013), as described in the previous subsection on the growth and mortality models. However, this coupling methodology can be used with all other growth and mortality models.

### Estimation and selection

We implemented a MCMC algorithm to estimate the parameters (Robert and Casella 2004). A random walk was used as proposal distribution to sample new values of parameters that were or were not selected, using the Metropolis–Hastings ratio. Only standard deviation *θ*_7_ was sampled in an inverse-gamma posterior distribution, using a Gibbs sampler. The functional traits used as demographical predictors were uncertain because botanical determination was incomplete for the older censuses, and not all values of functional traits were available for all species. We used the method developed in Aubry-Kientz et al. (2013) to handle these uncertainties. Because the growth and mortality models were created separately, some functional traits were independently selected in the two processes. We used the method proposed by Kuo and Mallick (1998) to select the most useful predictors in the joint model. The method then was applied to eight predictors: *Hmax*,*Ortho*,*WD*, and *Tough* of the mortality process and *DBH*95, *WD*,*Hmax*, and *δ*13*C* of the growth process.

All algorithms and statistical treatments were implemented using R software (R Core Team 2014). The R codes developed in this study are available in the Supplementary materials 1.

## Results

### Model inference

Starting from randomly chosen values, we realized 2000 iterations of the Metropolis–Hastings algorithm in order to achieve convergence of 100 parallel MCMC chains. Then, we reduced the variance of the proposition laws and reran the algorithm. A satisfying staying rate was achieved after 3500 iterations. We then used a burning of 1000 iterations and a thinning of 10 to achieve a satisfying autocorrelation. Parameters of the growth process converged slower (between 100 and 600 iterations) than parameters of the mortality process (between 10 and 100 iterations). This reflects the weight of each process in the total likelihood. The growth process influenced the two terms of the likelihood, while the mortality process influenced only the term linked with the probability of dying. The histogram resulting from the Kuo-Mallick selection procedure showed a large break with no values between 0.4 and 0.8 (Fig. 2). The 0.4 value was the indicator associated with orthotropic orientation (*Ortho*). Therefore, this predictor was not included in the final coupled model.

### Growth process

The growth process was adjusted by a sigmoid curve that can be biologically interpreted and that depends on tree size. Parameters *θ*_1_…*θ*_4_ link the maximum growth to the functional traits of each tree (Table2). These parameters have the same signs as in Hérault et al. (2011), meaning that maximum growth rates increased with increasing *DBHmax* and decreasing *Hmax*,*WD*, and *δ*13*C*. Diameter at maximum growth was attained for 0.767*DBH95_*s*_. The value of *θ*_6_ converged above 2, meaning that species with denser wood modulate their growth less (i.e., they have flatter growth trajectories).

**Table 2:** Results of the estimation method

Parameter	Predictor	Median	90% credibility interval
*β*_0_	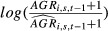	−0.403	[−0.446; −0.359]
*β*_1_	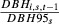	0.140	[−0.430; 0.625]
*β*_2_	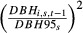	0.502	[0.175; 0.905]
*β*_3_	*Hmax*_*s*_	−0.414	[−0.463; −0.365]
*β*_5_	*WD*_*s*_	−0.951	[−1.24; −0.622]
*β*_6_	*Tough*_*s*_	−0.327	[−0.397; −0.254]
*θ*_1_	*DBH*95_*s*_	2.43	[1.98; 2.92]
*θ*_2_	*WD*_*s*_	−0.384	[−0.545; −0.246]
*θ*_3_	*Hmax*_*s*_	0.0318	[−0.0435; 0.103]
*θ*_4_	*δ*13*C*_*s*_	−0.403	[−0.467; −0.333]
*θ*_5_	*DBH*95_*s*_	0.767	[0.866; 3.304]
*θ*_6_	*WD*_*s*_	4.81	[3.422; 6.67]
*θ*_7_	*ɛ*	27.5	[27.0; 28.0]

For each parameter of the model, median, and 90% credibility interval of the posterior distribution. Note that *β*_4_ does not appear in the final model because *Ortho* was not selected by the selection procedure.

### Mortality process

**Figure 1:**
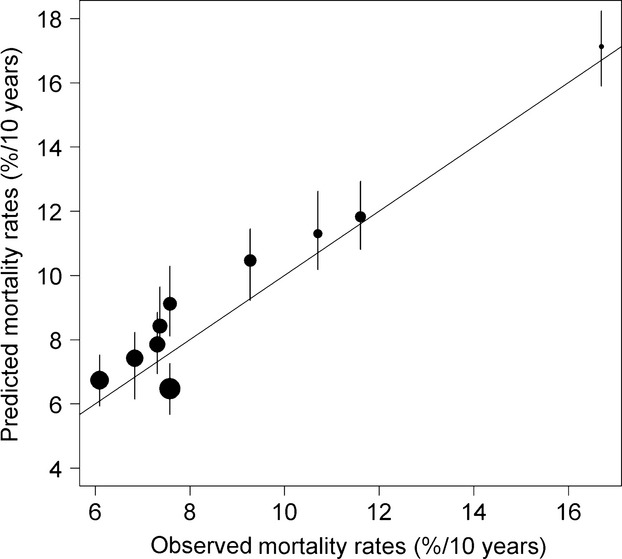
Mortality depends on tree vigor. Predicted mortality rates versus observed mortality rates. Trees were regrouped into 10 groups depending on the value of the vigor estimate. Circle sizes are proportional to the averaged vigor of the groups. As mortality is a stochastic process, 100 simulations were realized and predicted values are plotted. Segments correspond to the 90% credibility interval.

The mortality process depends on the vigor estimator, on two estimators of the ontogenetic trajectory, and on four functional traits: *Hmax*,*Ortho*,*WD*, and *Tough* (Table2). All parameters linking the functional traits and the probability of dying had the same sign as in Aubry-Kientz et al. (2013). This means that the probability of dying is even higher when the tree is small, has a low density of wood, or has fragile leaves (Fig. 3). The parameter *β*_0_ makes the link between the growth process and the mortality process thanks to the vigor estimator: 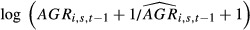. This parameter takes values around −0.4, implying that a tree with a higher past growth than expected will have a lower probability of dying (Fig. 1). The adjusted pseudo-r2 of Mac Fadden without vigor was 0.0288 and 0.0492 with vigor.

## Discussion

**Figure 2:**
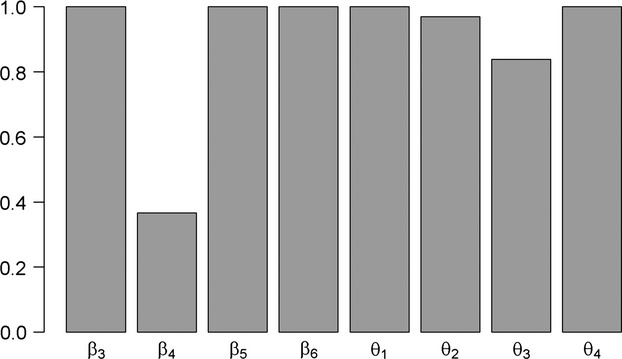
Results of the Kuo-Mallick algorithm for parameter selection. Median of the distribution for each variable; variables are included in the final model if the median value is inferior as 0.8. A gap with no value between 0.4 and 0.8 is observed, and the variable associated with *Ortho* (*β*_4_) takes value 0.4, which is why *Ortho* is not included in the final model. Variables included are as follows: *Hmax* (*β*_3_), *WD* (*β*_5_), *Tough* (*β*_6_) in the mortality process; and *DBH*95 (*θ*_1_), *WD* (*θ*_2_), *Hmax* (*θ*_3_), *δ*13*C* (*θ*_4_) in the growth process.

**Figure 3:**
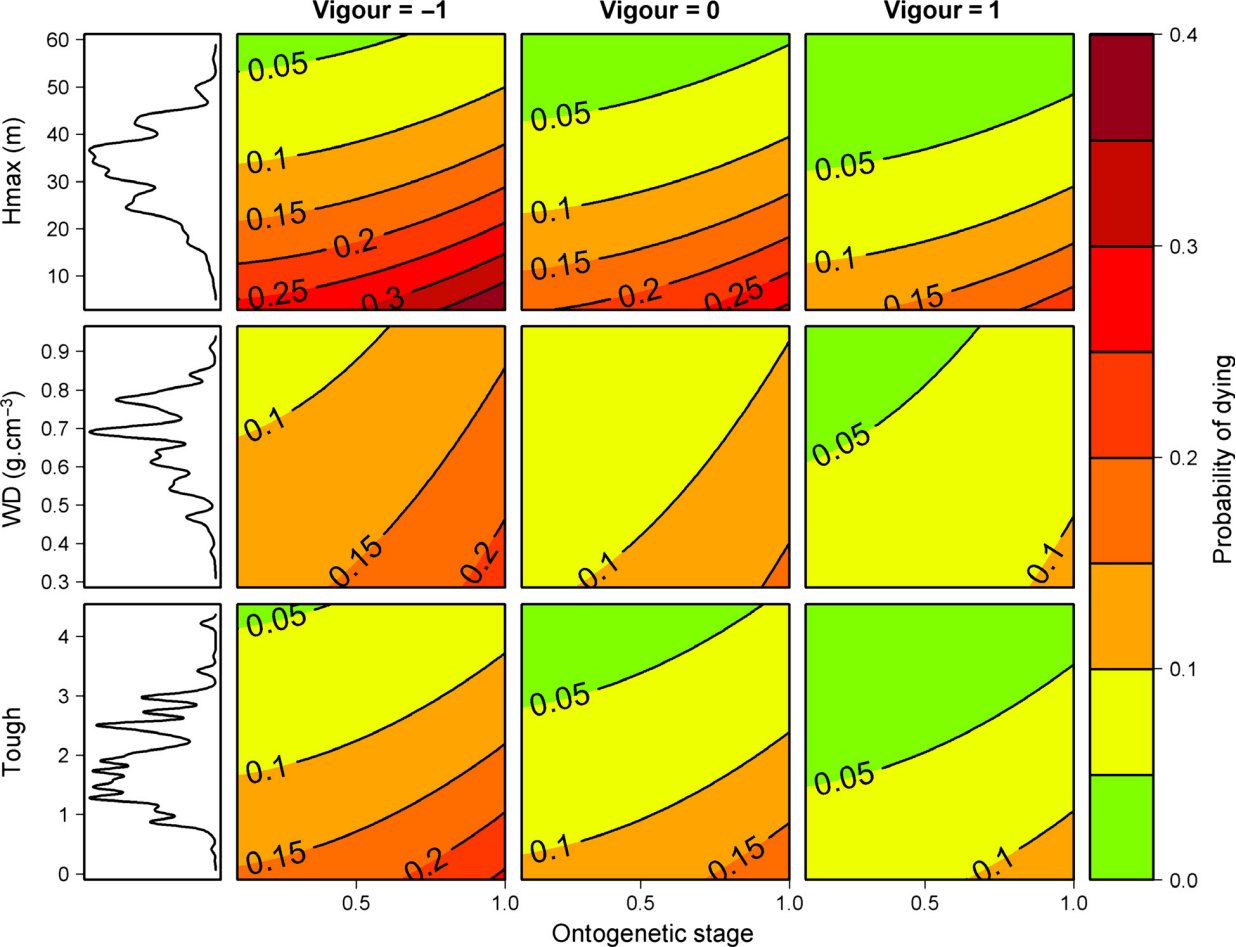
Tree vigor is a predictor of mortality. Simulations using the final mortality model. Tree lines correspond to tree functional traits used as predictors: *Hmax*,*WD*, and *Tough*. The three columns corresponds to tree vigor: −1, 0, and 1. Probability of dying is plotted, depending on the ontogenetic stage estimated by 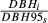 where *DBH*_*i*_ is the diameter of tree *i* and *DBH*95_*s*_ is the maximum diameter for species *s* to which tree *i* belongs to.

In this study, we modeled size-dependent growth and mortality of a tropical tree community, using a joint individual-based modeling framework. The posterior values obtained are coherent with results of Hérault et al. (2011) and Aubry-Kientz et al. (2013), increasing our confidence in the biological determinisms of the ecological processes we want to model. Moreover, this confirms that the functional trait-based approach could be successfully used to predict tree dynamics in highly diverse tropical forests for which dynamic data may be lacking, but functional trait data are available. The explicit link between functional traits and tree dynamic parameters bridges the gap between individual-based and community-level models with little parameter inflation (Fig. 3). Building a vigor estimator and including it in the mortality model allowed us to take into account individual variability, which previously was not possible. The introduction of vigor into the mortality model greatly improved the prediction and highlighted that vigor is a key driver of mortality (Figs. 2, 3). The Mac Fadden's adjusted pseudo-*R*^2^ can be interpreted as the ratio of the estimated information gain when the model is used in comparison with the null model in order to estimate the information potentially recoverable by including all possible explanatory variables (Shtatland et al. 2002). In our case, the *R*^2^ was almost double when vigor was included, which implies that the goodness of fit would be almost double if the vigor estimator was included. Thus, past tree growth, independent of ontogeny and species ecological strategies, was by far the first predictor of tree mortality. Reduced radial growth is then intimately linked to increased mortality rates (Wunder et al. 2008) and as already asserted by Dobbertin (2005), the most important characteristic for any potential vigor indicator is the comparison with a suitable reference. We show that the difference between the computed expected growth and the growth observed in the field, 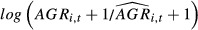, was an excellent candidate in hyperdiverse tropical forests (Fig. 1).

Individual tree growth was thus included in the mortality processes using the vigor estimator. The “easiest-to-implement” approach is to use directly the observed growth as a predictor in the mortality submodel (Chao et al. 2008; Metcalf et al. 2009; Rüger et al. 2011). However, this approach would not take into account the growth potential inherent in each species (Hérault, et al., [Bibr b15],[Bibr b16]) and would be at risk of generating double-counting in covariate selection. First, a low observed growth may be either due to a slow-growing species (in this case, the vigor will be close to 0) or, alternatively, to a slowdown in the growth of a fast-growing species due to an external forcing (in this case, the vigor will be negative). Second, the double-counting problem may be summarize in this way. Let *X* be a variable negatively impacting the growth of a tree. Because of the lower than expected induced growth, this tree will have greater risk of dying. If the growth and mortality models were not joint, then the variable *X* would be retained in the growth model and the mortality model, generating a double-counting of the specific effect of this variable. In our framework, the variable *X* will be retained in the growth model only and will be propagated in the mortality process through the vigor estimator. The parameter *β*_0_ associated with the vigor estimator was easily estimated, and all Markov chains converged quickly around −0.4. This demographical link between growth and mortality rates has already been well accepted (Dobbertin 2005; Chao et al. 2008) and is mainly related to changes in the carbon allocation of trees. Under environmental stress, trees are supposed to place higher priority on new foliage or new roots and lower priority on radial growth (Wunder et al. 2008). Slow-growing trees are thus likely to be unhealthy, exhibit physiological stress, and be prone to infection or death (Van Mantgem et al. 2003; Bigler et al. 2004). Most temperate-zone studies confirmed that tree vigor is a good indicator of mortality risk (Yao et al. 2001).

In our framework, an additive error describes the vigor of each tree individually, regardless of its species or ontogenetic stage. We first need to acknowledge that the vigor term did not separate out process error from observation error, and this may be a significant limitation for a correct ecological interpretation. This individual error is normally distributed and centered around 0 with standard deviation *θ*_7_. The vigor estimator was thus exactly the error of the growth model. This means that the posterior distribution of *θ*_7_ could be directly used to predict the probability of dying. For example, a tree with high value of the vigor estimator deterministically would have a higher growth rate and a smaller probability of dying. However, this is questionable when we want to use this joint model in a forest simulator. Roughly, there are two possibilities. Either an error is sampled at each time step, or each individual tree has its own vigor, which does not change throughout its life. This is an important choice in the simulation, and it is clear that the reality may be more nuanced. Even if the vigor of a tree may change during its life because of biotic or abiotic factors (Moravie et al. 1999), there is also increasing evidence of temporal dependence (Woollons and Norton 1990). Some studies indicated that the inclusion of temporal dependence in stochastic dynamic models results in outputs that are similar to those of deterministic models (Kangas, [Bibr b17],[Bibr b18]), but the theoretical and ecological basis of the stochastic model is clearly superior to that of the deterministic model (Fox et al. 2001). A more realist alternative could thus be to sample a new vigor estimator at each time *t*, depending on the vigor estimator at time *t*−1, such as in an autoregressive model (Suarez et al. 2004).

The available methods of combining nonlinear tree growth and mortality processes are not commonly used by forest ecologists. Our inference methodology is based on an MCMC approach, which allowed us to sample the model parameters according to their posterior distribution, using the joint model likelihood. This likelihood takes growth and mortality forest dynamic processes into account at each step of the algorithm. Hence, growth and mortality parameters are estimated simultaneously until stabilization. The joint model parameters were sampled individually to increase convergence speed. When the sampled parameter concerned the mortality process only, the growth process did not change and only the term of the likelihood linked to the growth process (*f*(*D*_*i*,*t*_¦*D*_*i*,*t*−1_)) was updated. When the estimated parameter concerned the growth process, as the growth process is plugged into the mortality process, all terms of the likelihood were affected: *f*(*D*_*i*,*s*,*t*_|*D*_*i*,*s*,*t*−1_) and *p*_*i*,*s*,*t*_. In practical terms, the parameters involved in the mortality process converged more easily than parameters involved in the growth process. Indeed, tree growth is strongly shaped by many additional environmental variables (*e.g*., topography, light availability, climate) (Hérault et al. 2010; Wagner, et al. [Bibr b44],[Bibr b45]) and by local competition (Uriarte et al. 2004; Laurans et al. 2014); however, neither factor was investigated in the present methodological study. Nevertheless, our methodology succeeded in binding the two processes, and the selection method allowed keeping only the useful variables as the parameters in the final model. Thus, the collinearity of the two processes was managed.

## Conclusion

In this study, we present a flexible methodology for building a growth-mortality joint model using the tree vigor. The chosen vigor estimator, based on the difference between observed and predicted growth, significantly improved by a factor 2 the accuracy of the mortality model predictions. This result confirmed that the individual behavior of trees is of great importance in the processes of growth and mortality and therefore should be much considered in tropical forest dynamic modeling studies. We successfully applied our conceptual framework to a complex hyperdiverse tropical forest community, taking into account different sources of data uncertainties. Our joint model methodology may be used with a wide range of growth and mortality submodels. Indeed, because the joint model likelihood is explicit, the inference process needs only one growth model that computes 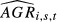 and a mortality model that computes *p*_*i*,*s*,*t*_. Once computed, these two sets of values are used to calculate the global likelihood and to estimate posterior distributions for the two submodels. The growth model may be more straightforward (e.g., a linear model) or more complex (e.g., random individual or species effects). This joint model approach provides an interesting framework for including other predicting variables possibly linked with tree dynamics, such as environmental and climatic variables. In the context of climate change, proper methodological frameworks are needed both to model the effect of climate variations on the dynamics of hyperdiverse tropical forest communities (Wagner et al. 2011; Choat et al. 2012; Feeley et al. 2012; Brienen et al. 2015) and to include these climate-explicit models into forest simulators in order to test their resilience to future expected changes.

## Supporting Information

Appendix S1 R codes
