# Supplementary material for: A joint individual-based model coupling growth and mortality reveals that tree vigor is a key component of tropical forest dynamics
Source: Ecol Evol. 2015 May 29;5(12):2457–65. doi: 10.1002/ece3.1532 (PMC4475377; doi:10.1002/ece3.1532)
Supplement: Appendix S1 — R codes [file ece30005-2457-sd1.pdf]

Supplementary Material

S1. R codes

We developed functions to allow an easy use of the computational method used in our study.

- *growth model*: a function specifying the growth model to be estimated.
- *mortality model*: a function specifying the mortality model to be estimated.
- *loglikelihood growth*: a function computing the part of the loglikelihood depending of the growth process.
- *loglikelihood mortality*: a function computing the part of the loglikelihood depending of the mortality process.
- *prep data*: a function to formalized the data create tables that will be used in the estimation process.
- *chain building*: a function to create the Markov chains for each parameter of the model.
- *plot function*: a function to plot the chains.
- *creation data*: a function to create simulated data.

The two models used in the articles are implemented and the simplest one can be tested easily with simulated data.

```
growth_model <- function(theta,traits,data_DBH)
{
  lAGR_pred <- t(theta[1,] * traits[,,'DBH95']*10 *
   exp(-0.5 *
   ((log( (data_DBH[,2])/(theta[2,]*traits[,,'DBH95']*10) )/( theta[3,] * traits[,,'WD'] ) )^2)))
  return(lAGR_pred)
}
```

```
growth_model <- function(theta,traits,data_DBH)
{
  lAGR_pred <- t(t(theta[1,] * t(traits[,,'DBH95']/100) +
  theta[2,] * t(traits[,,'WD']) + theta[3,] * t(traits[,,'Height']/10) +
  theta[4,] * t(traits[,,'Delta13C']/10) ) *
  exp( -0.5 *
  ((log((data_DBH[,2])/t(theta[5,]*t(traits[,,'DBH95']*10)))/(t(theta[6,]*t(traits[,,'WD']))))^2)))
  return(lAGR_pred)
}

mortality_model <- function(theta,rate,traits,data_DBH,nb_sample_traits)
{
  logit_p <- t(theta[5,] * traits[(1:nb_sample_traits),as.character(data_DBH[,1]),'DBH95']*10 +
   theta[6,] * t(rate))
  return(logit_p)
}

mortality_model <- function(theta,rate,traits,data_DBH,nb_sample_traits)
{
  logit_p <- t(theta[8,] * t(rate)) + t(theta[9,] *
  t((data_DBH[,3]/10)/t(traits[(1:nb_sample_traits),as.character(data_DBH[,1]),'DBH95']))) +
  t(theta[10,] *
  t(((data_DBH[,3]/10)/t(traits[(1:nb_sample_traits) ,as.character(data_DBH[,1]),'DBH95']))^2))+
  t(theta[11,] * (traits[(1:nb_sample_traits),as.character(data_DBH[,1]),'Height']/10)) +
  t(theta[12,] * (traits[(1:nb_sample_traits),as.character(data_DBH[,1]),'Ortho'])) +
  t(theta[13,] * (traits[(1:nb_sample_traits),as.character(data_DBH[,1]),'WD'])) +
  t(theta[14,] * (traits[(1:nb_sample_traits),as.character(data_DBH[,1]),'Tough']))
  return(logit_p)
}
```

```
loglikelihood_growth <- function(theta_sigma,AGR_obs,lAGR_pred,data1_unique,rep,nb_sample_traits)
{

  ll_growth <- dnorm(log(AGR_obs+1),mean=lAGR_pred,
            sd=matrix(theta_sigma,ncol=nb_sample_traits,nrow=length(AGR_obs),byrow=T),log=TRUE)
  rownames(ll_growth) <- rownames(data1_unique)

  ll_growth_tot <- colSums(ll_growth * as.double(rep))
  return(ll_growth_tot)
}

loglikelihood_mortality <-
function(theta,AGR_obs2,AGR_obs3,lAGR_pred2,lAGR_pred3,data2_unique,data3,nb_sample_traits,rep2)
{
  # living trees:
  rate1 <- log(AGR_obs2 + 1) - lAGR_pred2
  ll_morta_alive <- - log( 1 +
                    exp( mortality_model(theta,rate1,traits,data2_unique,nb_sample_traits) ))
  ll_morta_alive_tot <- colSums(as.double(rep2[rownames(data2_unique)]) * ll_morta_alive)

  # dead trees:
  rate2 <- log(AGR_obs3 + 1) - lAGR_pred3
  ll_morta_dead <- - log( 1 +
                   exp( - ( mortality_model(theta,rate2,traits,data3,nb_sample_traits) )))
  ll_morta_dead_tot <- colSums(ll_morta_dead)

  return(ll_morta_alive_tot+ll_morta_dead_tot)
}

prep_data <- function(data,CheckArguments = TRUE)
```

```
{
  if (CheckArguments)
  {
    check_arguments()
  }

  #### Creation of data1:
  ## DBH for living trees, used in the growth model

  #### Creation of data2:
  ## DBH for living trees, used in the mortality model

  #### Creation of data3:
  ## DBH for died trees, used in the mortality model

  id_alive <- rownames(data)[which(!is.na((rowSums(data))))]
  id_dead <- rownames(data)[which(is.na(data[,3]) & !is.na(data[,2]))]

  data1 <- data[as.character(id_alive),c(2,3)]
  data2 <- data[as.character(id_alive),c(1,2)]
  data3 <- data[as.character(id_dead),c(1,2)]

  for (i in 3:(ncol(data)-1))
  {
    data1 <- rbind(data1,data[as.character(id_alive),c(i,i+1)])
    data2 <- rbind(data2,data[as.character(id_alive),c((i-1),i)])

    id <- rownames(data)[which(is.na(data[,i+1]) & !is.na(data[,i]))]

    data3 <- rbind(data3,data[as.character(id),c(i-1,i)])
```

```
    for (j in 2:(i-1))
    {
      data1 <- rbind(data1,data[as.character(id),c(j,(j+1))])
      data2 <- rbind(data2,data[as.character(id),c((j-1),j)])

    }
  }

  data1 <- cbind(as.numeric(rownames(data1)),data1)
  data2 <- cbind(as.numeric(rownames(data2)),data2)
  data3 <- cbind(as.numeric(rownames(data3)),data3)

  return(list(data1=data1,data2=data2,data3=data3))

}

chain_building <- function(model,id_sigma,nb_iterations,nb_param,theta,nb_sample_traits,
step,traits,data_tree,A0,B0,mean_prior,sd_prior,CheckArguments = TRUE)

{
  if (CheckArguments)
  {
    check_arguments()
  }

  data1_unique <- unique(data_tree\$data1)
  data2_unique <- unique(data_tree\$data2)
  data3 <- data_tree\$data3
```

```
names1 <- unique(paste(as.character(data_tree\$data1[,1]),
     as.character(data_tree\$data1[,2]),as.character(data_tree\$data1[,3]),sep='_'))
rownames(data1_unique) <- names1
rep <- table(paste(as.character(data_tree\$data1[,1]),as.character(data_tree\$data1[,2]),
     as.character(data_tree\$data1[,3]),sep='_'))

names2 <- unique(paste(as.character(data_tree\$data2[,1]),
     as.character(data_tree\$data2[,2]),as.character(data_tree\$data2[,3]),sep='_'))
rownames(data2_unique) <- names2
rep2 <- table(paste(as.character(data_tree\$data2[,1]),as.character(data_tree\$data2[,2]),
    as.character(data_tree\$data2[,3]),sep='_'))

names3 <- paste(as.character(data3[,1]),as.character(data3[,2]),as.character(data3[,3]),sep='_')
rownames(data3) <- names3

names2_ok <- names2[which(names2\%in\%names1)]
names3_ok <- names3[which(names3\%in\%names1)]

AGR_obs <- apply(data1_unique[,c(2,3)],1,diff)
AGR_obs[AGR_obs<0] <- 0

AGR_obs2 <- apply(data2_unique[,c(2,3)],1,diff)
AGR_obs2[AGR_obs2<0] <- 0

AGR_obs3 <- apply(data3[,c(2,3)],1,diff)
AGR_obs3[AGR_obs3<0] <- 0

traits1 <- traits[(1:nb_sample_traits),as.character(data1_unique[,1]),]

lAGR_pred <- growth_model(theta,traits1,data1_unique)
```

```
rownames(lAGR_pred) <- names1

lAGR_pred2 <- matrix(NA,nrow=nrow(data2_unique),ncol=nb_sample_traits)
rownames(lAGR_pred2) <- names2
lAGR_pred2[names2_ok,] <- lAGR_pred[names2_ok,]
num2 <- which(is.na(lAGR_pred2[,1]))
traits2 <- traits[(1:nb_sample_traits),as.character(data2_unique[num2,1]),]
lAGR_pred2[num2,] <- growth_model(theta,traits2,data2_unique[num2,])

lAGR_pred3 <- matrix(NA,nrow=nrow(data3),ncol=nb_sample_traits)
rownames(lAGR_pred3) <- names3
lAGR_pred3[names3_ok,] <- lAGR_pred[names3_ok,]
num3 <- which(is.na(lAGR_pred3[,1]))
traits3 <- traits[(1:nb_sample_traits),as.character(data3[num3,1]),]
lAGR_pred3[num3,] <- growth_model(theta,traits3,data3[num3,])


llG <- loglikelihood_growth
      (theta[id_sigma,],AGR_obs,lAGR_pred,data1_unique,rep,nb_sample_traits)

llM <- loglikelihood_mortality
      (theta,AGR_obs2,AGR_obs3,lAGR_pred2,lAGR_pred3,data2_unique,data3,nb_sample_traits,rep2)

res <- array(dim=c(nb_iterations+1,nb_param,nb_sample_traits))
res[1,,] <- theta


for (compt in 1:nb_iterations)
{
```

```
ordre <- sample(1:nb_param)

for (i in ordre)
{
  theta_new <- theta

  if (i<id_sigma)
  {
    theta_new[i,] <- rnorm(nb_sample_traits,theta[i,],step[i])

    lAGR_pred_new <- growth_model(theta_new,traits1,data1_unique)
    rownames(lAGR_pred_new) <- names1

    lAGR_pred2_new <- matrix(NA,nrow=nrow(data2_unique),ncol=nb_sample_traits)
    rownames(lAGR_pred2_new) <- names2
    lAGR_pred2_new[names2_ok,] <- lAGR_pred_new[names2_ok,]
    lAGR_pred2_new[num2,] <- growth_model(theta_new,traits2,data2_unique[num2,])

    lAGR_pred3_new <- matrix(NA,nrow=nrow(data3),ncol=nb_sample_traits)
    rownames(lAGR_pred3_new) <- names3
    lAGR_pred3_new[names3_ok,] <- lAGR_pred_new[names3_ok,]
    lAGR_pred3_new[num3,] <- growth_model(theta_new,traits3,data3[num3,])



    llG_new <- loglikelihood_growth
         (theta_new[id_sigma,],AGR_obs,lAGR_pred_new,data1_unique,rep,nb_sample_traits)

    llM_new <- loglikelihood_mortality(theta_new,AGR_obs2,AGR_obs3,lAGR_pred2_new,
         lAGR_pred3_new, data2_unique,data3,nb_sample_traits,rep2)
```

```
  logr <- llM_new + llG_new - llG - llM +
       dnorm(theta[i,],theta_new[i,],step[i],log=T) -
       dnorm(theta_new[i,],theta[i,],step[i],log=T) +
       dnorm(theta_new[i,], mean_prior, sd_prior, log=T) -
       dnorm(theta[i,], mean_prior, sd_prior, log=T)

  r <- exp(logr)

  nb_ok <- which(!is.na(r))
  r_ok <- na.omit(r)

  accepte <- nb_ok[(runif(length(r_ok),min=0,max=1) < r_ok)]

  theta[,accepte] <- theta_new[,accepte]

  llG <- llG_new
  llM <- llM_new

  lAGR_pred <- lAGR_pred_new
  lAGR_pred2 <- lAGR_pred2_new
  lAGR_pred3 <- lAGR_pred3_new

}


if (i==id_sigma)
{

  theta[i,] <- rgamma(nb_sample_traits, A0+nrow(lAGR_pred)/2,
       (sum((log(AGR_obs+1)-lAGR_pred)^2))*0.5+B0)
```

```
  llG <- loglikelihood_growth(theta[id_sigma,],AGR_obs,lAGR_pred,
       data1_unique,rep,nb_sample_traits)

}

if (i>id_sigma)
{
  theta_new[i,] <- rnorm(nb_sample_traits,theta[i,],step[i])

  llG_new <- llG

  llM_new <- loglikelihood_mortality(theta_new,AGR_obs2,AGR_obs3,lAGR_pred2,lAGR_pred3,
       data2_unique,data3,nb_sample_traits,rep2)



  logr <- llM_new + llG_new - llG - llM +
      dnorm(theta[i,],theta_new[i,],step[i],log=T) -
      dnorm(theta_new[i,],theta[i,],step[i],log=T) +
      dnorm(theta_new[i,], mean_prior, sd_prior, log=T) -
      dnorm(theta[i,], mean_prior, sd_prior, log=T)

  r <- exp(logr)

  nb_ok <- which(!is.na(r))
  r_ok <- na.omit(r)

  accepte <- nb_ok[(runif(length(r_ok),min=0,max=1) < r_ok)]

  theta[,accepte] <- theta_new[,accepte]
```

```
        llM <- llM_new


      }

    }

    res[compt+1,,] <- theta

  }

  return(res)

}


plot_function <- function(result,burn,thin)
{

  nb <- dim(result)[2]
  l <- dim(result)[1]
  nb2 <- dim(result)[3]

  r <- nb%%3

  r2 <- 1
  if (r == 0)
    {r2 <- 0}
```

```
  a <- layout(matrix(c(1:(2*nb),rep(0,2*(3-r))),
   ncol = 2*((nb\%/\%3)+r2),nrow=3,byrow=T), heights=rep(1,3),
   widths = rep(c(1,0.2),((nb\%/\%3)+r2)))

  for (i in 1:nb)
  {
    par(mar=c(2,2,2,0.5))
    plot(result[seq(from=burn,to=l,by=thin),i,1],type='l',main=paste('theta',i))
    for (j in 2:nb2 )
    {
      lines(result[seq(from=burn,to=l,by=thin),i,j],col=j)
    }

    par(mar=c(2,0,2,1))
    plot(density(na.omit(result[seq(from=burn,to=l,by=thin),i,1]))\$y,
         density(na.omit(result[seq(from=burn,to=l,by=thin),i,1]))\$x,type='l',yaxt='n',xaxt='n')
    for (j in 2:nb2)
    {
      lines(density(na.omit(result[seq(from=burn,to=l,by=thin),i,j]))\$y,
           density(na.omit(result[seq(from=burn,to=l,by=thin),i,j]))\$x,type='l',col=j)
    }

  }

}

creation_data <- function(NbTree = 50, NbYears = 4, param = c(0.001,0.5,3,0.03,-0.004,-20),
param_sd = c(0,0,0,0,0,0),predictors = matrix(rnorm(NbTree*2,c(420,0.68),c(80,0.1)),ncol=2,byrow=T) ,
{
  if (CheckArguments)
```

```
{
  check_arguments()
}

epsilon <- rnorm(NbTree,0, param[4])
DBH <- matrix(rnorm(NbTree,15,0.2))

ID <- 1 : NbTree
names(epsilon) <- ID
rownames(predictors) <- ID
rownames(DBH) <- ID

dat <- DBH

DBH <- cbind(ID,DBH)

# growth function
growth_model <- function(id)
{
  lAGR_pred <- rnorm(1,param[1],param_sd[1]) * predictors[as.character(id),1] *
  exp( -0.5 *
  (( log( (DBH[as.character(id),2])/(param[2] * predictors[as.character(id),1]) )/
  ( param[3] * predictors[as.character(id),2] ) )^2) )
  return(lAGR_pred)
}

# mortality function
mortality_model <- function(id)
{
  logit_p <- rnorm(1,param[5],param_sd[5]) * predictors[as.character(id),1] +
```

```
     rnorm(1,param[6],param_sd[6]) * epsilon
    return(logit_p)
  }

  # first year:
  LC <- mortality_model(ID)
  p_death <- 1/( 1+exp(-LC))
  dead <- rbinom(NbTree,1,p_death)
  res <- rep(NA,NbTree)
  id_alive <- DBH[dead==0,1]
  lAGR_pred <- growth_model(id_alive)
  lAGR_sim <- lAGR_pred + epsilon[id_alive]
  res[dead==0] <- DBH[id_alive,2] + (exp(lAGR_sim)-1)
  dat <- cbind(DBH,res)

  for (i in 1:(NbYears-2))
  {
    dead <- rbinom(NbTree,1,p_death)
    res <- rep(NA,NbTree)
    names(res) <- rownames(dat)
    id_alive <- DBH[dead == 0 & !is.na(dat[,i+2]),1]
    lAGR_pred <- growth_model(id_alive)
    lAGR_sim <- lAGR_pred + epsilon[id_alive]
    res[id_alive] <- dat[id_alive,i+2] + (exp(lAGR_sim) - 1)
    dat <- cbind(dat,res)
  }

  dat <- dat[!is.na(dat[,2]),]

return(dat)
```

```
}
```
